# Development of a rat model of lymphedema and the implantation of a collagen-based medical device for therapeutic intervention

**DOI:** 10.3389/fcvm.2023.1214116

**Published:** 2023-07-04

**Authors:** Dung Nguyen, Dimitrios Dionyssiou, Tatiana S. Zaitseva, Anna T. Zhou, Gloria Sue, Peter Deptula, Maxim A. Moroz, Peter Tabada, Stanley G. Rockson, Michael V. Paukshto, Ming-Huei Cheng, Ngan F. Huang

**Affiliations:** ^1^ Department of Plastic and Reconstructive Surgery, Stanford University, Stanford, CA, United States; ^2^ Department of Plastic Surgery, Aristotle University of Thessaloniki, Greece; ^3^ Fibralign Corp, Union City, Thessaloniki, CA, United States; ^4^ Division of Plastic and Reconstructive Surgery, University of California San Francisco, San Francisco, CA, United States; ^5^ Department of Radiology, Stanford University, Stanford, CA, United States; ^6^ Division of Cardiovascular Medicine, Stanford University, Stanford, CA, United States; ^7^ A+ Surgery Clinic, Taipei, Taiwan; ^8^ Department of Cardiothoracic Surgery, Stanford University, Stanford, CA, United States; ^9^ Stanford Cardiovascular Institute, Stanford University, Stanford, CA, United States; ^10^ Center for Tissue Regeneration, Repair and Restoration, Veterans Affairs Palo Alto Health Care System, Palo Alto, CA, United States; ^11^ Department of Chemical Engineering, Stanford University, Palo Alto, CA, United States, United States

**Keywords:** lymphatic, lymphedema, scaffold, rat, animal model, method

## Abstract

Secondary lymphedema is a common condition among cancer survivors, and treatment strategies to prevent or treat lymphedema are in high demand. The development of novel strategies to diagnose or treat lymphedema would benefit from a robust experimental animal model of secondary lymphedema. The purpose of this methods paper is to describe and summarize our experience in developing and characterizing a rat hindlimb model of lymphedema. Here we describe a protocol to induce secondary lymphedema that takes advantage of micro computed tomography imaging for limb volume measurements and visualization of lymph drainage with near infrared imaging. To demonstrate the utility of this preclinical model for studying the therapeutic benefit of novel devices, we apply this animal model to test the efficacy of a biomaterials-based implantable medical device.

## Introduction

Lymphedema is a common side effect of cancer treatment with an overall incidence of 15.5%, and increased risk among patients who undergo pelvic dissections (22%) or radiation therapy (31%) (1–3). The average incidence of secondary lymphedema in women following breast cancer treatment is 20% (3) and a number of studies report an increased incidence of up to 60% (4–6). With sustainable treatment options remaining scarce, major efforts to reduce the incidence of lymphedema have been focused on early diagnostics, refinements in surgical techniques for cancer surgeries, and a combination of multiple imaging modalities used pre-, intra-, and postoperatively. A recent surgical approach to prevent lymphedema in high-risk patients, such as those undergoing axillary lymph node dissection (ALND), is lymphovenous bypass to an axillary vein tributary that is performed at the time of ALND (7–14). It decreases the rate of lymphedema down to as low as 4% (14, 15). In parallel, to treat symptomatic secondary lymphedema patients, microsurgical techniques such as vascularized lymph node transfer (VLNT) and lymphovenous anastomosis (LVA) have been increasingly used. The reported rates of volume reduction following VLNT or LVA are typically less than 60% (16) and underscore the need for further improvements in treating lymphedema.

To investigate effective management strategies for the treatment of lymphedema, a stable and reproducible animal model is needed. Lymphedema models usually involve relevant lymph node removal that may be coupled with radiotherapy. Physiologically, a porcine model approximate features of clinical lymphedema. Hindlimb lymphedema in pigs was induced by removal of superficial inguinal and popliteal lymph nodes followed by irradiation (17), and then evaluated by bioimpedance, contrast computed tomography (CT) and magnetic resonance imaging (MRI). The limitations of using this model include the high cost of animal maintenance, along with the availability of CT and MRI equipment. In addition, the thickness of the porcine skin prevents the use of indocyanine green (ICG) for intravital monitoring of lymphatics. Other models of lymphedema involve the removal of popliteal lymph nodes and adjacent lymphatic vessels in the hindlimbs (canine, sheep, and rabbit models) (18–20), and axillary lymph nodes in forelimbs (rat model) (21). The effect of irradiation prior to lymph node removal in hindlimbs was elegantly studied in mouse model (22). The removal of both deep and superficial lymphatic vessels around the circumference of the thigh muscle coupled with radiation therapy (45 Gy) in rat hindlimb induced a chronic lymphedema condition persisting up to 9 months (23), as evaluated by tape measurements of limb circumference and water-fill method, although this radiation dose was associated with considerable mortality. A lower dose of 20 Gy following removal of both inguinal and popliteal lymph nodes in rat hindlimb induced lymphedema with minimal morbidity in 4 months (24). Microcomputed tomography (microCT) imaging was used to precisely evaluate volumetric changes of lymphedematous and contralateral healthy limb, and lymphoscintigraphy to explore lymph drainage. The 20 Gy irradiation dose was adopted in a forelimb lymphedema study with a two-month follow-up (25). The hind limb location of the rat model is economically and technically reproducible, thus enabling the investigation of surgical treatments to combat chronic lymphedema. In addition, microCT imaging technology is available in many research centers, and this model has the advantage of using ICG imaging to detect lymph drainage patterns. For these reasons, the rat lymphedema model has become a well-accepted model of lymphedema.

In this methods paper, we describe and summarize our experience in generating and validating a rat hindlimb model of lymphedema. This protocol employs the established experimental design with microCT-based limb volume measurements, refines the visualization of lymph drainage with ICG imaging, and is used to evaluate the efficacy of therapeutic biomaterials and implantable medical devices (26–28) to improve the treatment of lymphedema.

## Materials and equipment

**Animals:**

Sprague-Dawley female rats (300 g, Charles River).

**General supplies:**

rat ear tag kit (Fine Science Tools).

hair removal cream (Nair).

bandage/ flexible wrap (generic brand).

povidone iodine (generic brand).

sterile gauze pads (generic brand).

alcohol prep pads (generic).

**Anesthetics, analgesia, and injections:**

Isoflurane (Isoflo; Abbott Laboratories).

lidocaine.

Analgesia (ie buprenorphine or carprofen).

Xylazine (Xylaject; Phoenix Pharmaceuticals or generic brand).

Ketamine (Ketaset; Fort Dodge Animal Health or generic brand).

Evans blue (Sigma–Aldrich).

Indocyanine Green (ICG) dye (MedChemExpress LLC).

**Surgical supplies:**

scalpels disposable # 15 (VWR).

cauterizer with bipolar tips (Fine Science Tools).

small scissors straight (Fine Science Tools).

small scissors curved (Fine Science Tools).

forceps flat (Fine Science Tools, CA).

forceps w/teeth (Fine Science Tools).

forceps thin (Fine Science Tools).

needleholder small (Fine Science Tools).

needleholder medium (Fine Science Tools).

GEM 1521 SuperFine MicroClips titanium hemostatic clips (Synovis MCA).

SuperFine MicroClip Applier (15 cm, Synovis MCA).

4-0 Prolene sutures (Ethicon).

9-0 ETHILON™ Nylon Suture 9-0 (Ethicon).

Michel Suture Clips—11 mm × 2 mm (Fine Science Tools).

Magnifying surgical glasses (individually fitted on prescription glasses) or alternatively, surgical microscope.

**Equipment:**

Surgical bench with anesthesia set-up with isoflurane and oxygen supplies and heating pad.

CT Scanner (TriFoil eXplore CT 120).

Polaris 225-MXR Mobile x-Ray System (Kimtron, Inc).

MXR-226 Unipolar x-Ray Tube (Comet Technologies USA, Inc).

Near-infrared PDE-NEO camera equipped with Swann HD Digital Video Recorder [or UR-4MD recorder (TEAC Corp)].

### Methods

The outline of procedures and timeline is summarized in Table 1.

**Table 1:** Procedure time point and duration details.

Time point (day)	Procedure	Procedure time per animal (min)
−1 or 0	MicroCT procedure	40
0	Removal of left inguinal and popliteal lymph nodes	45–60
	Device implantation as preventive approach.	10–20
10–14	Irradiation of the surgical site	40
30	MicroCT procedure	40
	Device implantation as treatment	20–30
60	MicroCT procedure	40
90/120	MicroCT procedure	40
	ICG injection; NIR imaging	30–45

## Results

### Pre-operative and post-operative micro-CT imaging

**Figure 1:**
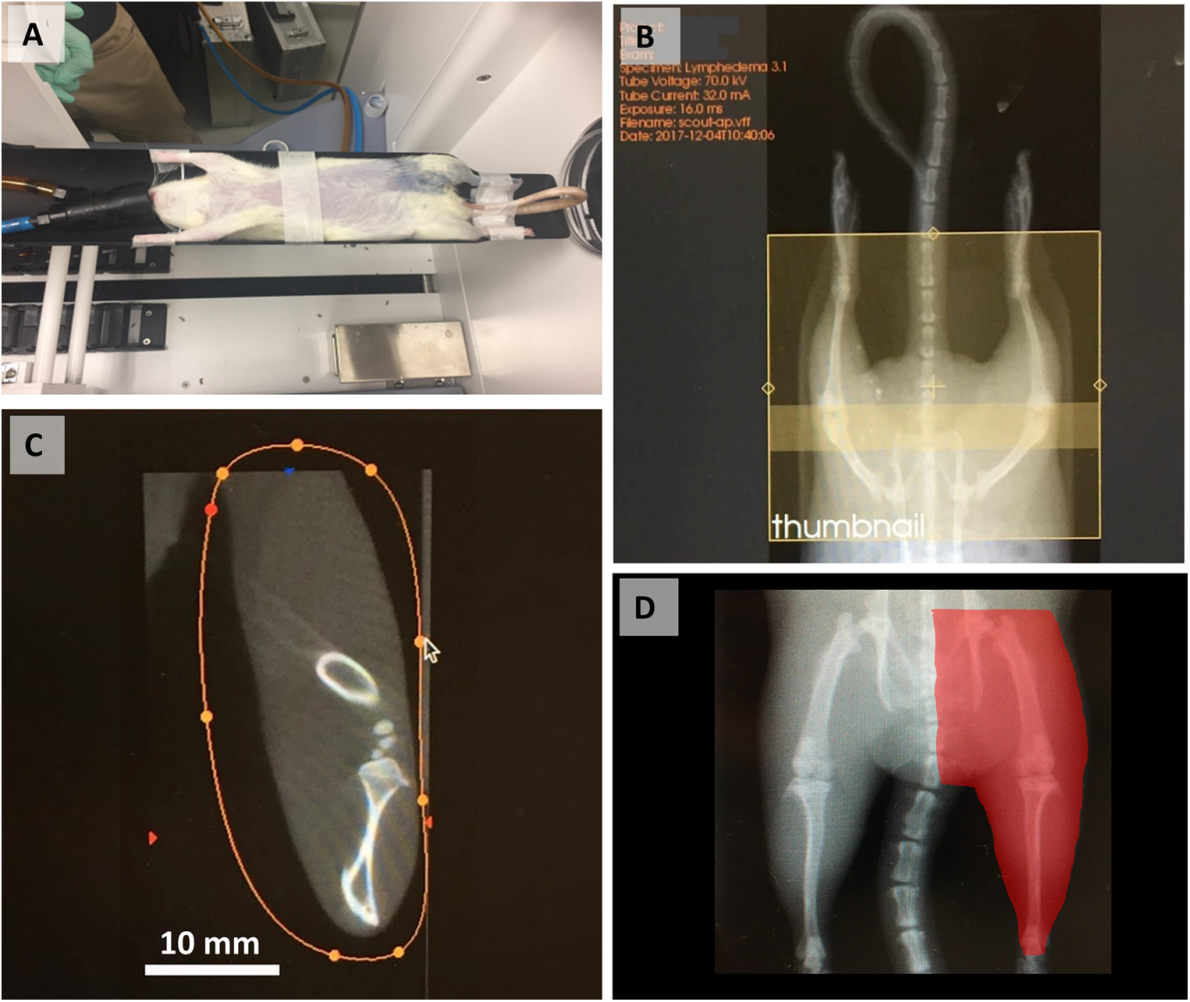
MicroCT procedure and image analysis steps. (**A**) Animal positioning in the scanner. (**B**) Selection of the scanning area from the femoral head to the ankle. (**C**) Selection and verification of the ROI by limb slice area. (**D**) Final calculated hindlimb volume (red highlighted area). Reproduced with permission (29).

MicroCT imaging and analyses were performed preoperatively and at 1-, and 3-months postoperatively by using microCT (TriFoil eXplore CT 120) (Figure 1). Volumetric analyses of the healthy and affected limbs were performed using the following protocol. After induction of anesthesia with 2%–2.5% isoflurane, female Sprague–Dawley rats (300 g) were transferred to the rat holder in microCT scanner. The animals were placed in the supine position and fit with the nose cone with inflow isoflurane and outflow evacuation tubing. Isoflurane inflow was turned on to maintain anesthesia. The rat's position with symmetrically full hind limb extension was secured with soft surgical tape (Figure 1A). After the scan area was set to span the femoral head to the ankle joint, the resultant 8-cm transaxial field of view encompassed both lower extremities (Figure 1B). A 15-min scan with 100 kVp energy, an exposure time of 1,000 ms, and 100 lA energy was performed. All images were reconstructed with filtered back-project into a three-dimensional image volume with pixel size of 0.2 mm in both the transverse and axial directions, by sequential selection and verification of the region of interest (ROI) that encompassed the limb slice area, for each image and for each hind limb (Figure 1C). All images were saved in DICOM format and analyzed with software (PMOD Technologies, Zurich, Switzerland). The volume of the hind limb was calculated from ankle joint to hip joint with reconstruction of transverse, sagittal and coronal sections (Figure 1D). The relative amount of excess volume (in %) was defined as the volume of the affected (left) limb minus the volume of the healthy (right) limb divided by the volume of the healthy (right) limb, multiplied by 100% (see the details in Data analysis section).

### Unilateral hind limb lymphedema rat model

Unilateral hind limb lymphedema was established in female Sprague–Dawley rats (300 g) based on a published protocol (24). All animal procedures were approved by the Institutional Animal Care and Use Committee at Stanford University.

**Figure 2:**
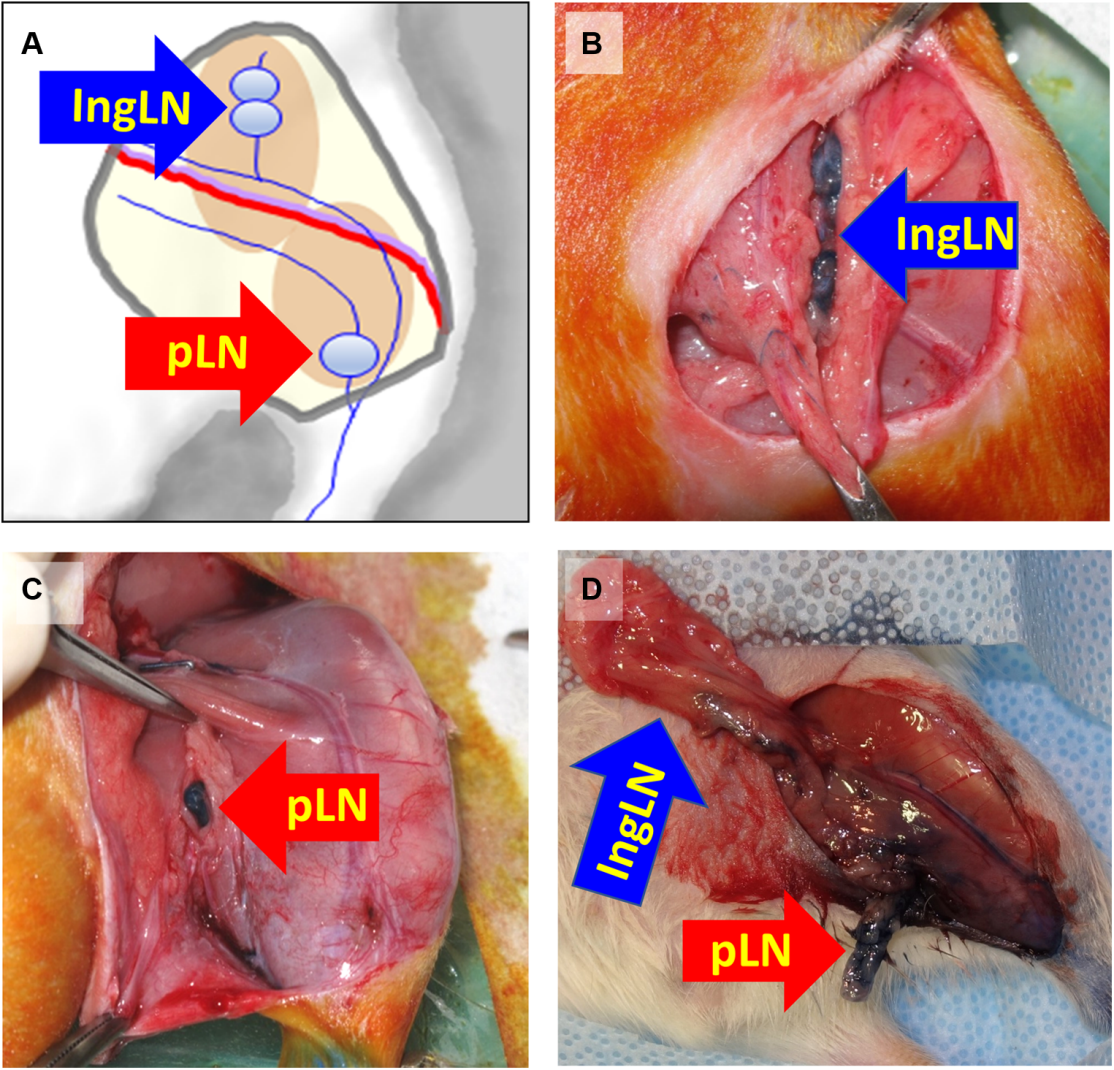
Schematics of mapping (**A**) and identifying the inguinal (**B**) and popliteal (**C**) lymph nodes after injection of Evans Blue in the paw in preparation (**D**) for lymph node excision. IngLN (inguinal lymph node); pLN (popliteal lymph node). Reproduced with permission (24).

General anesthesia was induced and maintained with 2.5% isoflurane. The surgical site spanning the inguinal region toward below the knee was shaved and wiped with 70% ethanol and iodine. The animals were placed in supine position onto a sterile surgical pad, with their limbs and tails secured with soft tape to ensure the correct position, followed by subcutaneous administration of lidocaine (2 mg/kg) for local pain control. An injection of 0.1 ml 10% Evans blue (Sigma–Aldrich, Saint Louis, MI) was made intradermally to the paw on the surgical site side. A full skin incision of 4–5 cm was made from inguinal area to a point below the knee, and inguinal and popliteal lymph nodes as well as accompanying lymphatic ducts were identified by absorbed blue stain (Figures 2A–D). Both inguinal and popliteal lymph nodes were excised, as well as thorough removal of the lymphatic tissue around the nodes, based on visualization of Evans blue–stained lymphatics (Figure 2) (24). Large vessels supplying lymph nodes were closed by applying microclips, and small afferent and efferent blood and lymphatic vessels were cauterized to minimize bleeding and loss of tissue fluids. Skin incisions were closed with metal clips. A soft sport bandage wrap was applied around the body from the forelimbs down to the inguinal area to prevent the animal from interfering with the wound. Buprenorphine at 0.05 mg/kg or carprofen at 5 mg/kg was administered post-operatively for at least 2 days.

### Therapeutic implantation of BioBridge collagen scaffolds

**Figure 3:**
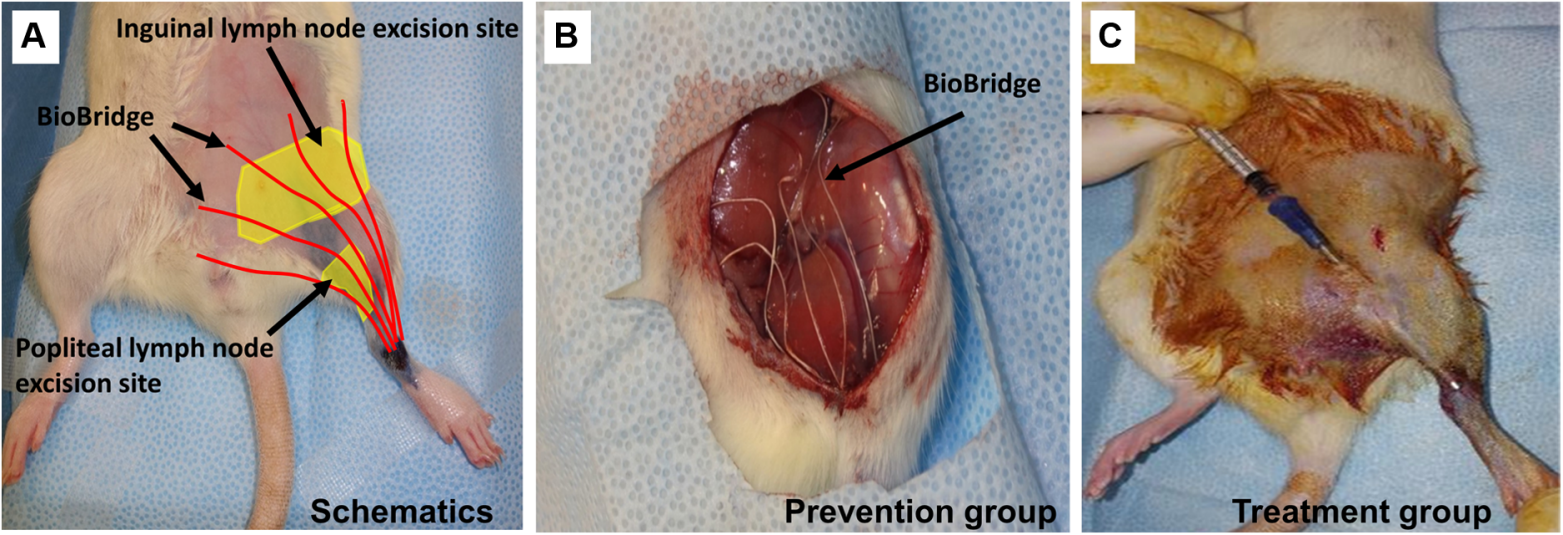
Schematic of BioBridge medical device implantation in a rat lymphedema model (**A**). Depiction of BioBridge collagen scaffold-based device (denoted by red color) implantation to bridge the area of obstruction from the lymph node excision site to the foot. (**B-C**). Specifically, it is shown as a preventive option where the devices are implantated immediately after lymph node resection (**B**), or as a treatment option after lymphedema has already developed (**C**). Reproduced with permission (27).

To demonstrate the utility of this disease model for translational applications, we then tested the efficacy of implanted nanofibrillar collagen scaffolds denoted as BioBridge scaffolds as a medical device to bridge the region of lymphedema. Implantation of BioBridge or other experimental therapies may be performed immediately after the lymph node removal surgery (preventive approach) or after development of lymphedema was confirmed by microCT-based limb volume analysis [treatment option (27)]. The general schematic of implantation and two suggested options of BioBridge implantation are shown in Figure 3.

### Lymphedema prevention model

At the end of the lymphedema initiation surgery, five ∼5-cm strands of BioBridge medical devices were implanted by placing one end below the knee and the other end toward the midline and/or the proximal border of inguinal area surgical site toward the ipsilateral proximal lymphosome border and then securing the devices (Figure 3B). Importantly, BioBridge ends should cross the midline/border by several mm in order to reach the area with healthy lymphatics. BioBridge can be attached to the surrounding connective or subcutaneous fat tissue with microclips or 9-0 nylon sutures. The attachments were done at the periphery of the scaffold to avoid occluding the central parallel channels. The wounds were closed with staples/clips.

### Lymphedema treatment model

**Figure 4:**
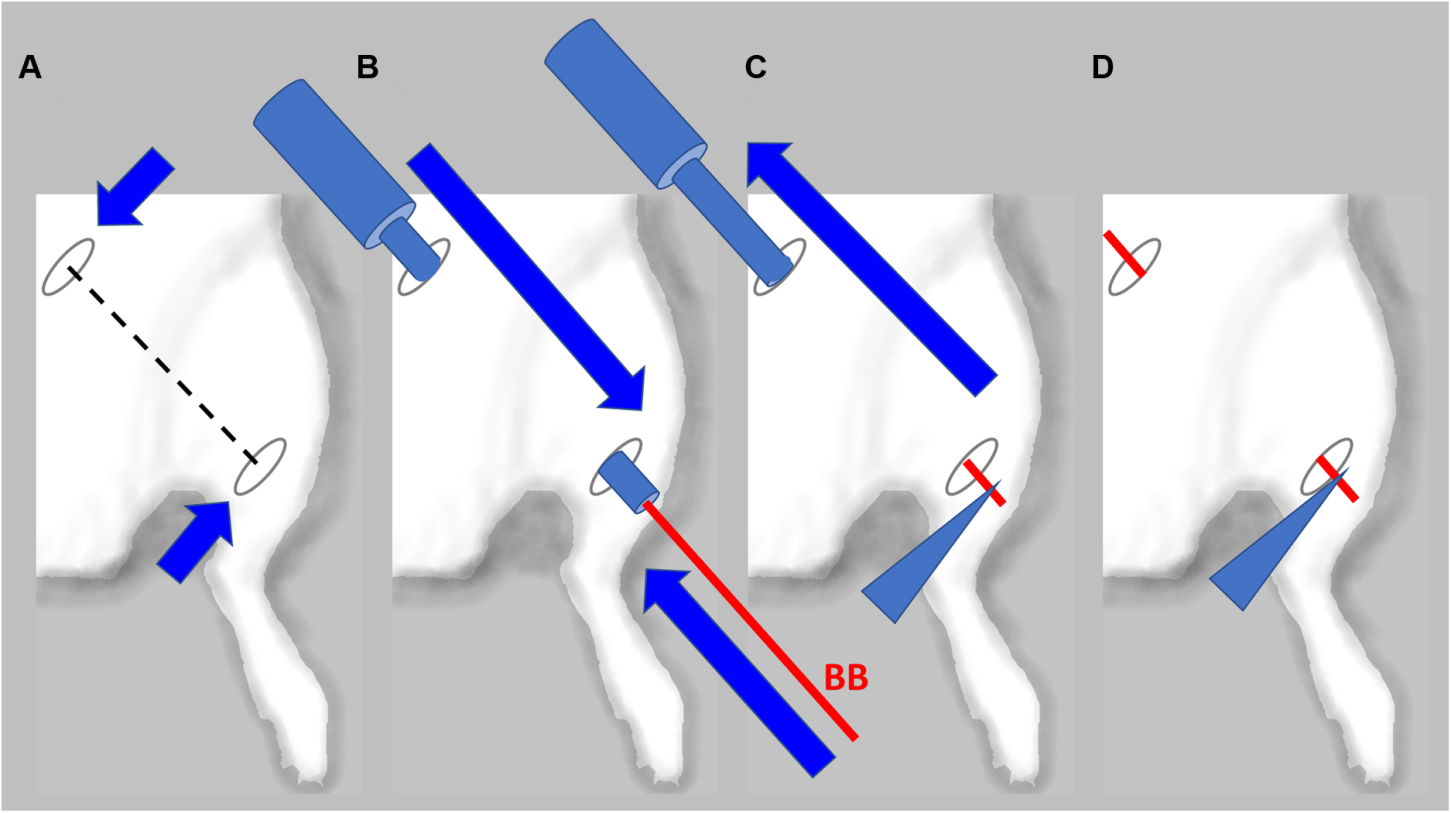
Detailed schematics of BioBridge medical device implantation in a rat lymphedema model. (**A**). Exit and entry incisions are made. (**B**). Trocar is inserted through the incisions, and BioBridge (BB) is inserted into the trocar opening. (**C**). BB is held in place with forceps, while trocar is being removed. (**D**). After the trocar was removed, the BB is left in place.

In animals with confirmed lymphedema (4–5 weeks after lymph node resection and irradiation), after induction of anesthesia, the animals receive implantations of five BioBridge devices following a similar design as the prevention model. Specifically, the devices were implanted towards the midline and/or toward the ipsilateral proximal lymphosome border or vascularized lymph node transfer. One approach to implant the devices is to perform an open surgery as in preventive option. The second approach is to use large gauge needles (18G to 14G) as trocars (Figure 3C, details in Figure 4). In the latter case, a small incision was made at the insertion and at the exit points (Figure 4A). The trocar was inserted under the skin through the insertion point and then pushed to the exit point. With a trocar in place, the BioBridge was then inserted into the trocar all the way, with its end left protruding from the trocar (Figure 4B). This protruding end was held in place, and the trocar was gently removed (Figure 4C), keeping the BioBridge implanted along the trocar-made path (Figure 4D). The punctured skin openings were closed with 5-0 chromic sutures to prevent scaffold migration. The advantage of using a trocar-like device for collagen scaffold implantation is that an additional treatment, such as cultured cells, can also be applied.

### Irradiation

Regardless of the choice of a prevention or treatment model, the animals were subjected to irradiation one week (±4 days) after lymph node resection, when the wounds have healed. The animals were anesthetized with ketamine (80 mg/kg) and xylazine (12 mg/kg) for radiotherapy. Once the animals were under deep anesthesia, they were placed on the work stand under the irradiator, and all body parts except for area intended to be irradiated were covered with lead shielding. Animal limbs and tails were secured with soft surgical tape where needed to ensure correct positioning. The surgery site was irradiated with a single dose of 20 Gy (Polaris 225-MXR Mobile x-Ray System; Kimtron, Inc.; Oxford, CT) using a MXR-226 Unipolar x-Ray Tube (Comet Technologies USA, Inc.; Shelton, CT). The dimensions of the radiation field were 3 × 4 cm with a depth of 1.5 cm.

### Allocation of animals to control and treatment groups

Lymphedema was confirmed at 4-5 weeks after lymph node resection when the affected hind limb had the volume increased by 5% or more vs. the normal hind limb. Hindlimb volume was calculated for the entire volume from ankle joint to hip joint by micro-CT imaging. Animals that did not show at least a 5% volume increase were excluded.

### NIR fluoroscopy

To visualize the lymphatics at higher resolution than Evans blue dye, ICG imaging was performed after the final microCT procedure. The animals were anesthetized with 2.5% isoflurane. A 0.1-ml aliquot of 3.3 mM ICG (MedChemExpress LLC, NJ, US) aqueous solution was injected intradermally into the paw, and the uptake of ICG was monitored by near-infrared PDE-NEO camera equipped with Swann HD Digital Video Recorder. Alternatively, a UR-4MD recorder may also be used (TEAC Corp) (27). The PDE-NEO camera was positioned 20 cm from the examination area and secured in place over the area of interest with a ring stand or another stable platform. To prevent signal interference, any ambient infrared light sources were turned off. Precise visualization of the fluorescence signal with respect to subject body anatomy was achieved by switching between color and fluorescence imaging modes. Near-focus camera mode was used to capture more minute details in the lymphatic system. Switching between color and fluorescence imaging modes allowed for precise visualization of the fluorescence signal location in the animals. After injection, the ICG signal intensified over time. To maintain image quality during this change, fluorescence controls for brightness and contrast were adjusted accordingly. Decreasing the brightness obviated oversaturation and loss of detail in animals requiring longer ICG signal monitoring. To view downstream lymphatic pathways, the brightness was increased to adjust for the decreased ICG signal. The duration of NIR fluoroscopy was approximately 30–45 min per animal.

**Figure 5:**
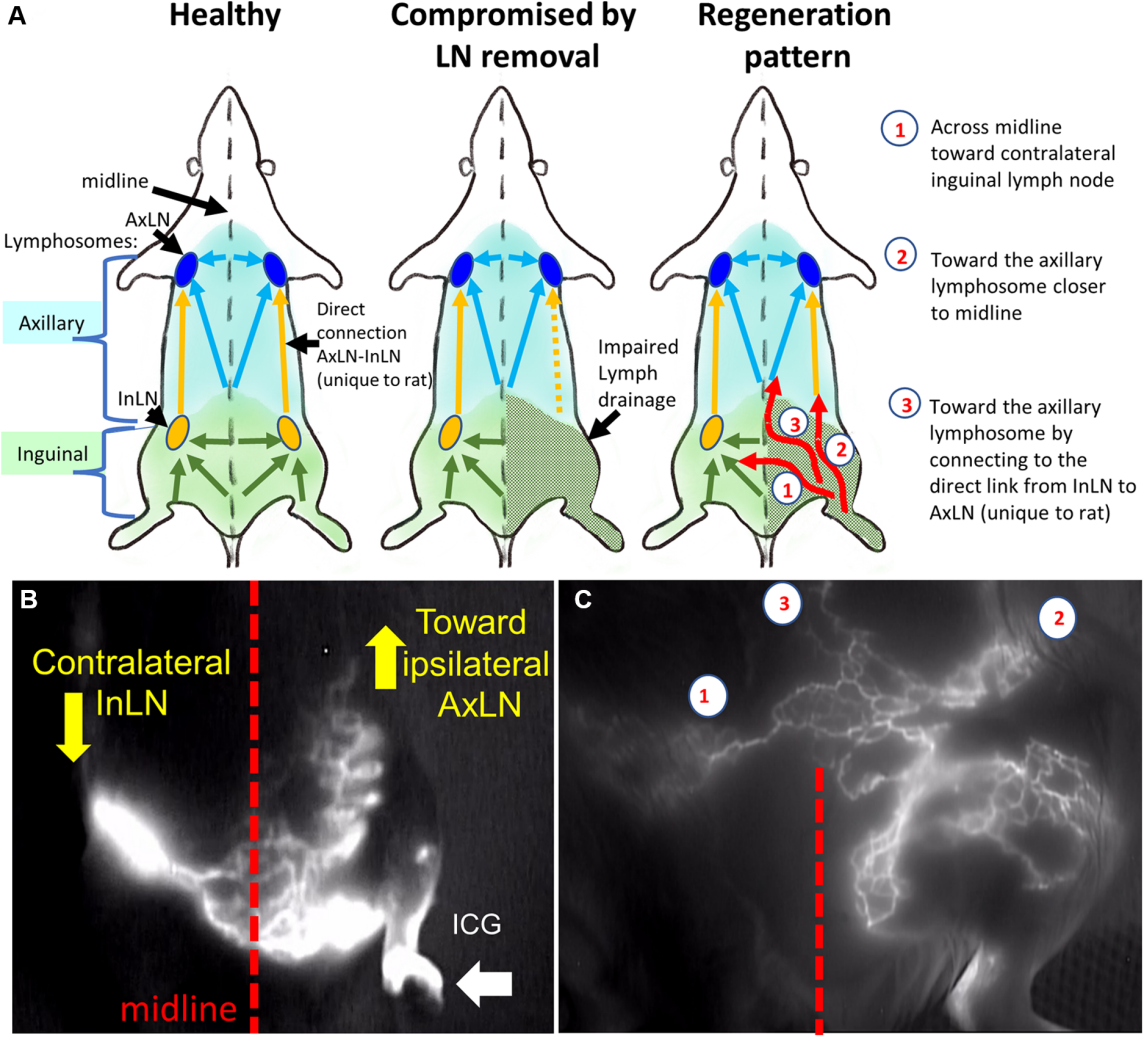
Rat lymphosomes and lymphatic vasculature patterns revealed by ICG imaging. (**A**). Schematics of healthy, compromised and regenerated lymphatic drainage patterns. (**B**) Lymphatics from affected area were re-routed toward ipsilateral axillary lymph node and contralateral inguinal lymph node. (**C**) the main re-routed directions were the (1) contralateral inguinal lymph node (InLN), (2) deep ipsilateral lymphatics, (3) ipsilateral axillary lymph node (AxLN). Reproduced with permission (27).

Imaging analysis at 3 months post-treatment involved the evaluation of lymphatics and mapping the lymphatics in the treated groin area using NIR imaging. The analysis steps included (1) quantifying the ICG propagation time to the midline or next closest lymphosome border; (2) counting the number of lymphatic collectors based on ICG staining; and (3) describing the pattern of the lymphatics as either tortuous, curvy, or straight. An example of post-treatment lymphatic vasculature patterns relative to the current understanding of the lymphosome map in the rat is shown in Figure 5.

### Data analysis

Clinical staging of lymphedema is based on volumetric measurements. In particular, the relative limb volume change (in %) or relative amount of excess volume (in %) is the ISL recommended and widely accepted characteristic of unilateral edema (30, 31). If A_1_ and U_1_ are the volumes of affected and healthy (unaffected contralateral) limbs at a first time point t_1_, A_2_ and U_2_ are the volumes of affected and healthy (contralateral) limbs at a second time point t_2_ then A_1_ – U_1_ is the volume of the edema at the first time point (e.g., before treatment) and A_2_ – U_2_ is the volume of edema at the second time point (e.g., after the treatment). Thus, relative amount of excess volume (in %) at the 1st time point is defined by the formula:

$$\left(\frac{A_{1}-U_{1}}{U_{1}}\right)100\text{\%}=\left(\frac{A_{1}}{U_{1}}-1\right)100\text{\%}$$

and relative amount of excess volume (in %) at the 2nd time point is defined by the formula:

$$\left(\frac{A_{2}-U_{2}}{U_{2}}\right)100\text{\%}=\left(\frac{A_{2}}{U_{2}}-1\right)100\text{\%}$$

The relative limb volume data make it possible to compare the edema at different time points. It is especially important to account for changes in animal weight during the study. Thus, the edema volume reduction and the rate of edema volume reduction can be measured by the respective formulas:

$$E_{r}=\left(1-\frac{\frac{A_{2}}{U_{2}}-1}{\frac{A_{1}}{U_{1}}-1}\right)100\text{\%}\;\;\mathrm{and}\;\frac{E_{r}}{t_{2}-t_{1}}.$$

Another metric for quantifying unilateral lymphedema, accounting for both the asymmetry of upper extremities' volumes and their temporal changes has been developed in (32). It defines the relative volume change as:

$$RVC=\left(\frac{A_{2}}{U_{2}}\right)/\left(\frac{A_{1}}{U_{1}}\right)-1$$

There is a similarity between E_r_ and RVC but the latter measure has no simple physical meaning.

Since volumetric clinical staging of lymphedema doesnot always correlate with the functional characterization based on ICG fluoroscopy (33), it is important to take into consideration both criteria. Therefore, lymphatic drainage should be assessed quantitatively by ICG fluoroscopy for number and morphology of new collectors and for the time required for ICG to move from injection point to the middle line.

Some complementary data can be obtained using bioimpedance measurement, contrast CT lymphography, and dielectric constant measurements (17, 26).

## Discussion

Secondary lymphedema accounts for 99% cases of lymphedema and is most frequently presented in patients with flilariasis or cancer survival patients who underwent lymphadenectomy and irradiation treatment (34). A translational model of secondary lymphedema is key to optimizing current treatments and developing new approaches.

This model combines lymph node removal and irradiation to mimic the condition often occurring in cancer patients. This model causes lymphedema in 81.5% operated animals (24), provides a long-term condition of lymphedema which remains unresolved at 4 months after lymph node removal, and allows for feasibility to evaluate a range of surgical and microsurgical treatments with the portable equipment that surgeons routinely use in clinical setting (i.e., prescription magnifying glasses, NIR camera). A modification of this model omitting the irradiation resulted in lowering incidence of lymphedema by 46.7% (28).

The key step in the generation of the lymphedema condition, and its long-term maintenance is the meticulous excision of the lymph nodes and surrounding lymphatic vessels at the time of induction surgery. Failure to excise surrounding lymphatics may preclude development of lymphedema. Post-operatively, it is critical to monitor the animals daily after the surgery to prevent them from interfering with the wound. It is important to apply irradiation procedure once the wounds are healed to prevent the dehiscence later.

We use adult female Sprague-Dawley rats (300 g) since the primary goal of the model is to provide a condition that simulates lymphedema condition in women. Sprague-Dawley are outbred rats widely used to develop animal models of multiple human conditions (35). The fact that using a genetically heterogeneous outbred rodents in the development of animal models of human disease may introduce variation in disease manifestation as well as response to therapeutic intervention may be considered either as an advantage since these aspects are characteristic to human population as well, or a disadvantage if the variations are overwhelming.

The described lymphedema model in rat hindlimb causes an increase in limb volume that is not resolved spontaneously up to 4 months from the induction of lymphedema. This timeframe provides an opportunity to evaluate translationally relevant treatment modalities including VLNT, LVA, medical devices, cell therapy, and their combinations. To date, we are aware of only two medical devices that have been evaluated using this model and proceeded to the clinical studies. The first medical device is BioBridge (Fibralign Corp, CA), which has demonstrated the improvement or prevention of lymphedema in a rat model, as well as shown efficacy as an adjunct treatment in pilot clinical studies (27, 29), and is undergoing clinical trial for using in conjunction with VLNT (NCT04606030). The second device is a lymph drainage device (LymphoDrain, Lymphatica Medtech SA) that is based on mechanical pumping of excess lymph out of an affected area, which showed a decrease in limb volume in this rat model (26) and is currently undergoing a pilot clinical study (NCT04858230). The BioBridge is scaffold is expected to biodegrade within one year after implantation.

Employing the described model can potentially be extended to other biomaterial devices or scaffolds that have been shown to induce and support lymphangiogenesis (36). Devices designed to repair interrupted lymphatic drainage are usually intended to work as a three-dimensional template for cellular proliferation, matrix deposition, and structural organization with the ultimate goal of restoration of functional lymphatic channels (36). They include scaffolds made from biodegradable biopolymers (i.e., polyglycolic acid, fibrin, collagen) with specific biochemical and topographic features or matrices obtained by decellularization of materials produced from donor or cadaver tissue, which have the native tissue microarchitecture conserved. Decellularized scaffold formats were demonstrated to support lymphatic vessel growth, both alone (37) and in combination with cellular components (38, 39). All of the above can be evaluated in the current model, provided their dimensions are tailored to fit the missing lymphatic routes. Lymphangiogenesis can also be stimulated by other bioengineering constructs such as cell-only constructs or sheets (40) or silicone microgroove templates (25). If scaled up, these materials can be potentially evaluated in the current lymphedema model.

## Conclusion

The described hindlimb rat lymphedema model has been successfully used for verification and optimization of medical devices. It combines the benefits of clinical similarity (precise volume estimate, clear threshold of lymphedema diagnostics of 5% in excess volume, and comparable ICG imaging pattern) with high lymphedema incidence rate of about 80% and verified disease window of about 4 months.

## Data Availability

The raw data supporting the conclusions of this article will be made available by the authors, without undue reservation.
